# Effect of *Azolla* feeding on the growth, feed conversion ratio, blood biochemical attributes and immune competence traits of growing turkeys

**DOI:** 10.14202/vetworld.2018.459-463

**Published:** 2018-04-11

**Authors:** Mayank Shukla, Amitav Bhattacharyya, Pankaj Kumar Shukla, Debashis Roy, Brijesh Yadav, Rajneesh Sirohi

**Affiliations:** 1 Department of Poultry Science, College of Veterinary Science and Animal Husbandry, Mathura, Uttar Pradesh, India; 2 Department of Animal Nutrition, College of Veterinary Science and Animal Husbandry, Mathura, Uttar Pradesh, India; 3 Department of Veterinary Physiology, College of Veterinary Science and Animal Husbandry, Mathura, Uttar Pradesh, India; 4 Livestock Production and Management, College of Veterinary Science and Animal Husbandry, Mathura, Uttar Pradesh, India

**Keywords:** *Azolla*, biochemical attributes, body weight, immunity, turkeys

## Abstract

**Background and Aim:**

In the recent past,few studies have been carried out in chicken to assess the effect of *Azolla* meal and raw *Azolla* feeding on the performance of chicken. If turkeys effectively use unconventional feedstuffs like *Azolla* without reducing the performance, it will increase the profitability of turkey business. Hence, a study was carried out to evaluate the effect of dried *Azolla pinnata* vis-a-vis raw *Azolla* as choice feeding on the growth, feed conversion ratio (FCR), blood biochemical attributes, and immune competence traits of growing turkeys under intensive system.

**Materials and Methods:**

A total of 72, 8-week-old grower turkey poults of black variety were randomly distributed into three dietary treatments having three replicates each with eight birds. The birds of the control group (T1) were fed a basal diet (CP - 19.71% and ME - 2789.79 Kcal/kg), while the other group (T2) and choice-feeding group (T3) were fed 5% of basal diet replaced by dry *Azolla* powder on DM basis and *ad libitumAzolla* along with basal diet, respectively.

**Results:**

There was no significant difference among the different groups in the average weekly weight gain during the entire experiment. FCR was significantly better (p<0.05) in the choice-feeding group compared to the other two experimental groups during 8-16 weeks of age. There was no significant difference among the treatment groups in any of the blood biochemical indices except plasma uric acid, which was significantly decreased (p<0.01) in T2 compared to T1 at 16 weeks of age. HA and IgM response to 1% sheep red blood cells (log2 titer) were numerically better in T2 and T3 compared to the T1.

**Conclusion:**

Thus, it may be inferred that choice feeding with *Azolla*, and basal diet may improve FCR without any adverse effect on blood biochemical attributes and immune competence traits.

## Introduction

Research on the utilization of green forages and feed has increased considerably during the past few years. It has been seen that turkeys consume more vegetables (green feeds) than do poultry. Hence, feed factor is quite significant for turkeys under the intensive system, as they have no direct contact with plant feeds, especially green feeds. Besides, the phenomenal increase in poultry production has resulted in competition with the conventional human food ingredients leading to a shortage and increased the cost of conventional feed ingredients [1]. Since the cost of the feed accounts for nearly 75% of the total cost of turkey production, the substitution of conventional feedstuffs by unconventional feedstuff will lead to a reduction in the cost of turkey feed, and hence, increase the margin of profit in turkey business.

*Azolla* is a small aquatic fern that flows on the water surface. The use of *Azolla* as a drug, reclaiming saline soils [2], and bioremediation [3,4] has also been investigated. Few studies have been carried out in chicken to assess the effect of *Azolla* meal and raw *Azolla* feeding on the performance of chickens [5-7]. If turkeys effectively use unconventional feedstuffs like *Azolla* without reducing the performance, it will increase the profitability of turkey business.

It has already been established that choice-feeding system may play a pivotal role in reducing the feed cost of laying hens and turkeys in developing countries [8]. The basic principle behind free choice feeding is that individual birds can select from the various feed ingredients and thus get a chance to compose their diet according to their actual requirement and capacity of production.

Hence, the present study was carried out to evaluate the effect of dried *Azolla pinnata* meal vis-a-vis raw *Azolla* as choice feeding on the body weight, feed conversion ratio (FCR), blood biochemical attributes, and immune competence traits of growing turkeys under intensive system.

## Materials and Methods

### Ethical approval

Experiment was carried out in accordance with the guidelines laid down by the Institute Animal Ethics Committee for the use of poultry birds.

### Preparation of Azolla meal

Raw *A. pinnata* was procured from “*Azolla* Demonstration Unit” of the University. Raw *Azolla* was sundried in a clean and dust-free environment to obtain fine powder. The powder formed was packed in an airtight container.

### Experimental design, housing, feeding, and management

A total of 72, 8-week-oldblack variety ofturkey poultswere divided into three treatment groups, comprising three replicates and eight turkey poults in each replicate. The chicks were wing banded, weighed individually, and distributed randomly on uniform body weight basis in the treatment groups. The birds were housed in deep litter system. Water was offered *adlibitum*. The birds of the control group (T1) were fed a basal diet (19.71% CP and 2789.79 Kcal ME/kg), while the other group (T2) and choice-feeding group (T3) were fed 5% of basal diet replaced by dry *Azolla* powder on DM basis and *ad libitumAzolla* along with the basal diet, respectively. Representative samples of *Azolla* meal, T1 and T2,were analyzed for their nutrient composition, namely, dry matter, ether extract, crude protein, total ash, crude fiber, calcium, and phosphorous (Table-1).

**Table-1:** Proximate analysis of *Azolla* meal and turkey feed on dry matter basis.

Category	Dry matter (%)	Total Ash (%)	Ether extract (%)	Calcium (%)	Phosphorous (%)	Crude protein (%)	Crude fiber (%)
*Azolla* meal	98.8	21.67	3.15	1.11	0.59	25.64	17.29
T1	89.96	3.29	4.31	0.89	0.52	19.30	3.91
T2	85.95	3.85	4.25	1.41	0.79	19.67	4.58

### Body weight gain and FCR

Weekly body weight gain was recorded, and thereafter, FCR was determined for different phases of growth (8-12 weeks, 12-16 weeks, and 8-16 weeks) on the basis of feed consumption and on the basis of total dry matter intake (feed and *Azolla*).

### Biochemical attributes

After 16 weeks of age, blood was collected from the wing vein of 9 birds of each group (3 birds from each replicate) with the help of heparinized and non-heparinized syringes and poured into sterile tubes. The blood samples were centrifuged for the 10-15 min at 2500 rpm. Plasma and serum were separated and stored in refrigerator (−20°C) until analyzed. Plasma cholesterol, high-density lipoprotein cholesterol, protein, uric acid, glutamate oxaloacetate transaminase or aspartate aminotransferase, glutamate pyruvic transaminase or alanine aminotransferase, and alkaline phosphatase were determined using commercial kits (Span Cogent Diagnostics product, India), according to the manufacturer’s instructions.

### ELISA

Reactive oxygen species level in plasma was determined by ELISA using the method suggested by Brambilla *et al*. [9] with slight modification. 200 µL acetate buffer (0.1 M pH 4.8) was dispensed in 96-well plates (Nunc-Immunoplates, Nalge Nunc Italia, Rome, Italy) by a multichannel pipette. An aliquot of 5 µL for each serum, previously diluted 1:10 v/v, was then added by micropipette. Each sample was tested in duplicate. The plate was shaken gently for 1 min on a thermo-shaker (Gerhardt, Bonn, Germany). Then, 5 µL of a chromogen solution containing 0.37 M of N, N-diethyl-para-phenylenediamine as substrate was added to each well. Plates were incubated for 75 min at +37°C in the dark with gentle shaking. A standard curve was prepared using different concentrations of H_2_O_2_ in place of serum. Absorbance was read on a microplate reader (Bio-Rad ELISA reader) at 495 nm and values after background subtraction plotted against H_2_O_2_ concentrations. Serum superoxide dismutase (SOD) activity was measured using the method as described by Madesh and Balasubramanian [10] with some modifications. In the microtiter plate method, the assay mixture in a total volume of 300 µL per well consisted of 120 µL PBS, 10 µL serum sample, 5 µL of 1.25 mM 3-(4,5-dimethyl-thiazol-2-yl) 2,5-diphenyl tetrazolium bromide (MTT), and 15 µL of freshly prepared 1 mM pyrogallol solution to be added at the end. Sample was replaced with PBS in the blank. After an incubation period of 15 min, 150 µL of dimethyl sulfoxide was added and absorbance was taken in ELISA reader at 570 nm. The percentage inhibition by the presence of SOD was calculated from the reduction of the MTT color formation as compared to the MTT formazan formed in the absence of SOD which was taken as 100%.

### Immune competence traits

After 16 weeks of age, general immune response was studied by taking 9 birds from each treatment group (3 birds from each replicate) and measuring important immunocompetence traits such as antibody response (log_2_ titer) to 1% sheep red blood cells (SRBC) [11,12], 2-mercaptoethanol resistant antibodies (MER or IgG) and mercaptoethanol-sensitive antibodies (MES or IgM) against SRBC [13], and cell-mediated immune response to phytohemagglutinin-P (PHA-P) [14].

### Statistical analysis

Data were subjected to one-way analysis of variance in a completely randomized design [15]. Significant differences among treatment means were calculated as per Duncan’s multiple range test [16]. Differences among treatments were considered to be statistically significant when p≤0.05.

## Results

### Body weight gain

There was no significant difference in body weight gain among the treatment groups during 8-16 weeks of age (Table-2).

**Table-2:** Effect of *Azolla* feeding on the average weekly body weight gain (g) of grower turkeys during 8-16 weeks of age.

Weeks	Control	5% *Azolla*	Choice feeding	Pooled SEM	Significant level
9	179.50	167.92	181.79	4.41	NS
10	160.17	170.00	181.41	6.24	NS
11	183.13	160.34	137.50	8.49	NS
12	150.46	176.13	184.34	9.49	NS
13	208.46	225.63	218.92	6.54	NS
14	218.08	180.67	187.46	9.89	NS
15	206.26	195.58	189.96	6.93	NS
16	189.51	234.75	235.50	11.97	NS

NS=Non-significant(p>0.05) SEM=Standard error of means

### FCR

FCR was significantly better (p<0.01) in the choice-feeding group compared to the other two experimental groups during 8-12 weeks of age (Table-3). Further, FCR was numerically better in the choice-feeding group compared to the other two experimental groups during 12-16 weeks of age. In addition, overall FCR was significantly better (p<0.05) in the choice-feeding group compared to the other two experimental groups during 8-16 weeks of age. However, there was no significant difference observed in the overall FCR on the basis of total dry matter intake among the different treatment groups during 8-16 weeks of age (Table-3).

**Table-3:** Effect of *Azolla* feeding on the FCR (only feed) and FCR on total DM intake basis (feed and *Azolla*) of grower turkey during 8-12, 12-16, and 8-16 weeks of age.

Phases of growth (week)	FCR (only feed)					FCR on total DM intake basis (feed and *Azolla*)				
	
	Control	5% *Azolla*	Choice feeding	Pooled SEM	Sig. level	Control	5% *Azolla*	Choice feeding	Pooled SEM	Sig. level
8-12	2.86^a^	2.64^a^	2.34^b^	0.08	p<0.01	2.57	2.37	2.60	0.05	NS
12-16	3.51	3.62	3.24	0.09	NS	3.16	3.26	3.28	0.07	NS
8-16	3.21^a^	3.18^a^	2.83^b^	0.08	P<0.01	2.89	2.86	2.97	0.04	NS

Means bearing different superscripts within a column differ significantly (p<0.05). NS=Nonsignificant (p>0.05), SEM=Standard error of means, FCR=Feed conversion ratio

### Blood biochemical attributes

There was no significant difference among the treatment groups in any of the blood biochemical indices except plasma uric acid at 16 weeks of age (Table-4). Plasma uric acid was significantly decreased (p<0.01) in T2 compared to T1. However, there was no significant difference in the plasma uric acid between T2 and T3.

**Table-4:** Effect of *Azolla* dfeeding on blood biochemicals (protein, uric acid, cholesterol, AST, ALT, and ALP) of grower turkeys at 16 weeks of age.

Parameters	Control	5% *Azolla*	Choice feeding	Pooled SEM	Significant level
Protein (g/dl)	2.73	2.66	3.12	0.15	NS
Uric acid (mg/dL)	6.28^a^	5.42^b^	5.87^ab^	0.12	p<0.01
Cholesterol (mg/dL)	126.51	130.43	117.95	3.49	NS
AST (IU/L)	3.31	5.08	2.65	0.65	NS
ALT (IU/L)	7.74	5.30	5.30	0.74	NS
ALP (IU/L)	219.84	151.36	250.52	25.50	NS
HDL (mg/dL)	55.74	54.14	54.20	2.13	NS
SOD (units/mL)	216.56	218.01	219.59	2.11	NS
ROS (mg H_2_O_2_ equivalents/mL)	40.85	39.45	39.83	0.35	NS

Means bearing different superscripts within a column differ significantly (p<0.05). NS=Non-significant (p>0.05) SEM=Standard error of means, FCR=Feed conversion ratio

### Immune response

HA and IgM response to 1% SRBC (log_2_ titer) was numerically better in both the *Azolla*-fed groups compared to the control group (Table-5). Further, the HA and IgM response to 1% SRBC was comparatively better in the choice-feeding group compared to the 5% *Azolla*-fed group. However, IgG response was significantly higher (p<0.01) in the control group compared to the other two *Azolla*-fed groups.

**Table-5:** Effect of *Azolla* feeding on the humoralimmune responses (antibody titer [log 2] values) to 1% SRBC of values and cell-mediated immune response (response to PHA-P) (foot web index) of grower turkey at 16 weeks of age.

Parameters	Control	5% *Azolla*	Choice feeding	Pooled SEM	Significant level
HA	7.34	7.71	8.63	0.29	NS
IgG	1.50^a^	1.00^b^	1.00^b^	0.08	p<0.01
IgM	5.83	6.71	7.63	0.32	NS
Foot web index	0.61	0.76	0.78	0.05	NS

Means bearing different superscripts within a column differ significantly (p<0.05). NS=Nonsignificant (p>0.05), SEM=Standard error of means

No significant difference was found in response to PHA-P among the treatment groups (Table-5). However, cell-mediated immune response to PHA-P was numerically better in both the *Azolla*-fed groups compared to the control group.

## Discussion

### Chemical composition of Azolla

The proximate composition of *Azolla* was in concurrence with the values obtained in other studies [17-19]. The percent ether extract on dry matter basis of *Azolla* meal was 3.15, which was in agreement with the values 3.70 and 3.62 as reported by Balaji *et al*. [17] and Ara *et al*. [19], respectively. Similarly, the percentage of protein on dry matter basis of *Azolla* meal was 25.64, which was in concurrence with the values 24.5 and 26.02 as reported by Balaji *et al*. [17] and Parthasarathy *et al*. [18], respectively. Further, the percentage of total ash on dry matter basis of *Azolla* meal was 21.67, which was in concurrence with 18.1 reported by Ara *et al*. [19]. Similarly, the percentage of crude fiber on dry matter basis of *Azolla* meal was 17.29 and was in agreement with the values 14.3 and 14.9 reported by Ara *et al*. [19] and Balaji *et al*. [17], respectively.

### Growth performance

In our study, there was no significant difference in the body weight gain among the treatment groups. Similarly, Parthasarathy *et al*. [18] reported no significant difference in body weight gain of broilers on basal and 5% *Azolla* diets. Balaji *et al*. [17] also noted that inclusion of *Azolla* up to 4.5% in rations did not have any influence on body weight gain in broiler chicken. These observations suggested that green *Azolla*, when fed *ad libitum* with the basal diet or replaced 5% of the basal diet on DM basis, had no adverse effect on body weight gain. Further, Naghshi *et al*. [20] reported that chicken fed 5% *Azolla* powder had significantly (p<0.01) better daily weight gain compared to the basal diet.

In our study, the significantly better (p<0.01) FCR in the choice-feeding group compared to the other two experimental groups during 8-12 weeks of age and 8-16 weeks of age may be attributed to the intrinsic mechanism of the birds to choose the best food for them.

### Blood biochemical parameters

In the present study, values of biochemical parameters are in range as reported in other studies on turkeys [21]. There was no significant difference among the treatment groups in any of the blood biochemical indices except plasma uric acid at 16 weeks of age. Sujatha *et al*. [7] also noted that there was no significant difference in the serum protein concentration between the control group fed basal diet and *Azolla*-based diet in Nicobari fowls. The significantly lower plasma uric acid in the 5% *Azolla* group and numerically lower uric acid value in the choice-feeding group compared to the control group may be due to better utilization of protein in these two groups. However, there is a paucity of information on studies carried out in blood biochemical attributes about *Azolla*-based diets.

### Immune response

There was no significant difference in HA and IgM response to 1% SRBC (log_2_ titer) among the treatment groups. However, HA and IgM response to 1% SRBC (log_2_ titer) was numerically better in both the *Azolla*-fed groups compared to the control group. This is in agreement with the study of Bhattacharyya *et al*. [5] who also reported that total immunoglobulins and MES (IgM) antibody titer (log 2) values in response to SRBC were significantly higher (p<0.05) in the basal diet replaced with dry *A. pinnata* powder at 5.5% on dry matter basis birds than the control group commercial birds at 6 weeks of age. However, Sujatha *et al*. [7] noted that there was no significant difference between the mean HI titer and MER titer between the control group and fresh *Azolla* fed at 200 g per chick per day from 45 to 60 weeks in Nicobari fowls. Further, the HA and IgM response to 1% SRBC was comparatively better in the choice-feeding group compared to the 5% *Azolla*-fed group. This may be due to the reason that growing turkeys are good foragers and there is an intrinsic mechanism of the birds to choose the best food for them resulting in numerically better immunity.

The results of our present study revealed that there was no adverse effect of *Azolla* on the immune system of turkeys. Our results are in conformity with Bhattacharyya *et al*. [22] who also reported that antibody response to SRBC was not affected by green berseem replacement in the conventional ration of turkeys.

There was no significant difference in the cell-mediated response to PHA-P among the treatment groups. This is in agreement with the study of Sujatha *et al*. [7] who also noted that there was no significant difference in the cell-mediated immune response between the *Azolla*-fed group and the control group. However, cell-mediated immune response to PHA-P was numerically better in both the *Azolla*-fed groups compared to the control group. This may be due to the reason that turkeys are good foragers and access to green feeds plays an important role in determining the health and immunity of turkeys along with an intrinsic mechanism of the birds to choose the best food for them, resulting in better immunity. Bhattacharyya *et al*. [22] also reported that foot web index of grower turkeys was numerically higher in the choice-feeding group having access to *ad libitum* basal diet and green berseem. In another study, Bhattacharyya *et al*. [5] reported that foot index was significantly (P<0.05) higher in the basal diet replaced with dry *A. pinnata* powder at 5.5% on dry matter basis group birds than the control group commercial birds at 6 weeks of age.

## Conclusion

It may be inferred that choice feeding with *Azolla* and basal diet may improve FCR without any adverse effect on blood biochemical attributes and immune competence traits.

## Authors’ Contributions

PKS, AB, and DR designed the study. MS conducted the experimental work and RS assisted during the experiment. AB and BY drafted the manuscript and corrected it. All authors read and approved the final manuscript.
